# The Identification of Critical m^6^A RNA Methylation Regulators as Malignant Prognosis Factors in Prostate Adenocarcinoma

**DOI:** 10.3389/fgene.2020.602485

**Published:** 2020-12-04

**Authors:** Jiaju Xu, Yuenan Liu, Jingchong Liu, Tianbo Xu, Gong Cheng, Yi Shou, Junwei Tong, Lilong Liu, Lijie Zhou, Wen Xiao, Zhiyong Xiong, Changfei Yuan, Zhixian Chen, Di Liu, Hongmei Yang, Huageng Liang, Ke Chen, Xiaoping Zhang

**Affiliations:** ^1^Department of Urology, Union Hospital, Tongji Medical College, Huazhong University of Science and Technology, Wuhan, China; ^2^Department of Pathogenic Biology, School of Basic Medicine, Huazhong University of Science and Technology, Wuhan, China

**Keywords:** Ncpsdummy6-methyladenosine, prostate adenocarcinoma, prognostic signature, methyltransferase, biomarker, RNA methylation, LASSO Cox regression, consensus clustering

## Abstract

RNA methylation accounts for over 60% of all RNA modifications, and N^6^-methyladenosine (m^6^A) is the most common modification on mRNA and lncRNA of human beings. It has been found that m^6^A modification occurs in microRNA, circRNA, rRNA, and tRNA, etc. The m^6^A modification plays an important role in regulating gene expression, and the abnormality of its regulatory mechanism refers to many human diseases, including cancers. Pitifully, as it stands there is a serious lack of knowledge of the extent to which the expression and function of m^6^A RNA methylation can influence prostate cancer (PC). Herein, we systematically analyzed the expression levels of 35 m^6^A RNA methylation regulators mentioned in literatures among prostate adenocarcinoma patients in the Cancer Genome Atlas (TCGA), finding that most of them expressed differently between cancer tissues and normal tissues with the significance of *p* < 0.05. Utilizing consensus clustering, we divided PC patients into two subgroups based on the differentially expressed m^6^A RNA methylation regulators with significantly different clinical outcomes. To appraise the discrepancy in total transcriptome between subgroups, the functional enrichment analysis was conducted for differential signaling pathways and cellular processes. Next, we selected five critical genes by the criteria that the regulators had a significant impact on prognosis of PC patients from TCGA through the last absolute shrinkage and selection operator (LASSO) Cox regression and obtained a risk score by weighted summation for prognosis prediction. The survival analysis curve and receiver operating characteristic (ROC) curve showed that this signature could excellently predict the prognosis of PC patients. The univariate and multivariate Cox regression analyses proved the independent prognostic value of the signature. In summary, our effort revealed the significance of m^6^A RNA methylation regulators in prostate cancer and determined a m^6^A gene expression classifier that well predicted the prognosis of prostate cancer.

## Introduction

Prostate cancer (PC), namely prostate adenocarcinoma (PRAD), is a great threat to the male reproductive system (Attard et al., 2016). Although most prostate cancers are slow growing, it may spread to other parts of the body, as well as the primary tumor growing quickly. According to cancer statistics from American Cancer Society, PC is the second leading cause of death among American men with cancer, with an estimate of 191,930 new cases and 33,330 deaths, accounting for 21 and 10% of all sites, respectively (Siegel et al., 2020). Surgical removal of the prostate, radiation, chemotherapy, or hormonal therapy are recommended to treat most PC patients. Five-year survival rate of localized or regional PC patients is a whisker away from a hundred percent, while that of distant PC patients is as low as around 30% (Siegel et al., 2020). Notably, a quarter of PC patients are recurrent in 5 years (Schwartz et al., 2014), which may indicate an unfavorable outcome. Between 2013 and 2017, mortality rates appeared to have stabilized (Siegel et al., 2020). Hence, researchers are striving for a breakthrough in complicated biological processes and molecular mechanisms of PC to identify novel targets for treatment of PC patients.

RNA modification participates in many biological processes in vital movement, counting regulation of RNA post-transcriptional stability (Wang et al., 2014), localization (Fustin et al., 2013), translocation, slicing (Molinie et al., 2016) and translation (Meyer et al., 2015). There exists expanding evidence of its importance in tumor development and malignant progression, thus it has stolen the spotlight from many researchers. Up until now, over 100 distinct chemical modifications have been identified from RNAs containing mRNA, tRNA, miRNA, long non-coding RNA, etc. (Wang et al., 2014; Liu et al., 2016; Roundtree et al., 2017a; Boccaletto et al., 2018; Yang D. et al., 2018; Yang Y. et al., 2018; Du et al., 2019; Shi H. et al., 2019). Among all of these, RNA methylation accounts for over 60% in RNA modifications. Transcriptome-wide m^6^A mapping has been disclosed to provide the landscape of m^6^A RNA methylation for its crucial function in cellular differentiation, cancer progression and other processes.

M^6^A modification mainly occurs on adenine in RRACH (R = G, A; H = A, C or U) sequence, the functions of which are regulated by an expanding list of writers, readers and erasers (Fu et al., 2014; Zhao et al., 2017; Yang Y. et al., 2018; Sun et al., 2019; Zaccara et al., 2019). The m^6^A-writer-complex is also known as methyltransferase, including METTL3, METTL14, WTAP and KIAA1429; erasers like ALKBH5 and FTO as demethylase can reverse methylation; m^6^A is recognized by m^6^A binding proteins, i.e., m^6^A readers, including YTH domain proteins (YTHDF1, YTHDF2, YTHDF3, YTHDC1, and YTHDC2) and nuclear heterogeneous proteins HNRNP family (HNRNPA2B1 and HNRNPC) (Zaccara et al., 2019).

M^6^A is thought to be closely linked to various cancer types, including gastric cancer (Yue et al., 2019), colorectal carcinoma (Linnebacher et al., 2010), PC (Machiela et al., 2012), thyroid cancer (Heiliger et al., 2012), breast cancer (Kaklamani et al., 2011; Machiela et al., 2012; Akilzhanova et al., 2013; Long et al., 2013; Reddy et al., 2013), pancreatic cancer (Pierce et al., 2011; Lin et al., 2013), kidney cancer (Ortega et al., 2003; Jin et al., 2012), sarcoma (Hesser et al., 2018; Tan and Gao, 2018), leukemia (Casalegno-Garduno et al., 2010), etc. With the explosion of research, we may see a profound impact of m^6^A on the proliferation of cancer cells.

In PC, literatures link pathogenesis and progression of the tumor with m^6^A regulators that include METTL3 (Cai et al., 2019; Li et al., 2020; Ma et al., 2020; Yuan et al., 2020), VIRMA (Barros-Silva et al., 2020), YTHDF2, YTHDF3 (Li et al., 2018), and FTO (Melnik, 2015). To the best of our knowledge, it lacks a comprehensive analysis of the expression of m^6^A RNA methylation regulators in PC with clinicopathological characteristics, malignant progression, and prognosis.

Herein, we comprehensively investigated the role of RNA m^6^A modification in PRAD. First, a genome-wide study showed significant alteration in m^6^A RNA modification-related genes. Then the expression profile of 35 genes in the PRAD cohort with a normal cohort in the Cancer Genome Atlas (TCGA) database was explored. Afterward, consensus clustering was conducted according to the gene expression levels of m^6^A RNA methylation regulators with principal components analysis (PCA), survival analysis, Gene Ontology (GO), and Kyoto Encyclopedia Genes and Genomes (KEGG) for evaluation. Finally, we formed an efficient prognosis indicator comprising five pivotal genes related to m^6^A RNA methylation by the last absolute shrinkage and selection operator (LASSO) Cox regression.

## Materials and Methods

### Dataset

The data were gathered from TCGA project^1^, cBioPortal for Cancer Genomics^2^, and UCSC Xena browser^3^, including gene expression datasets (RNA-seq) on PRAD patients, as well as corresponding demographic (age and gender), clinicopathological (clinical M stage, clinical T stage, Gleason score, pathologic T stage, and pathologic N stage) and survival [overall survival (OS) and disease-free survival (DFS)] information. Anatomic stage or prognostic group was determined by American Joint Committee on Cancer (AJCC) Cancer Staging Manual 8th edition (2017) (Amin et al., 2017a,b; Buyyounouski et al., 2017). Patients without survival information were eliminated from further evaluation.

### Screening of m^6^A RNA Methylation Regulators

35 m^6^A RNA methylation regulators were selected from articles and reviews manually (Sun et al., 2019; Zaccara et al., 2019; Table 1). Corresponding RNA-Seq data were extracted from the TCGA-PRAD cohort for further study.

**TABLE 1:** The components of m^6^A RNA methylation regulators in writer-, reader- and eraser-complex.

	Regulators
Writers	KIAA1429 (VIRMA), METTL3, METTL14, WTAP, RBM15, RBM15B, METTL16, ZC3H13, and PCIF1
Readers	TRMT112, ZCCHC4, NUDT21 (CPSF5), CPSF6, CBLL1 (HAKAI), SETD2, HNRNPC, HNRNPG (RBMX), HNRNPA2B1, IGF2BP1, IGF2BP2, IGF2BP3, YTHDC1, YTHDF1, YTHDF2, YTHDF3, YTHDC2, SRSF3, SRSF10, XRN1, FMR1 (FMRP), NXF1, and PRRC2A
Erasers	FTO, ALKBH5, and ALKBH3

### Genetic Alterations and Screening of Differentially Expressed m^6^A RNA Methylation Regulators

Genetic alterations were gathered from the TCGA and PRAD project in cBioPortal. Differential expression profiles of m^6^A RNA methylation regulators were analyzed by “limma” package (Law et al., 2014; Ritchie et al., 2015; Phipson et al., 2016) with a cut-off value of *p* < 0.05 and visualized by several R packages thereafter. Also, correlation analysis was performed by R 4.0.2.

### Identification of Subgroups With a Distinct Prognosis Using Consensus Clustering

All PC patients were divided into subgroups by consensus clustering according to the expression of differentially expressed m^6^A RNA methylation regulators, utilizing the “ConsensusClusterPlus” package (Monti et al., 2003; Wilkerson and Hayes, 2010). Survival analysis, GO, and KEGG pathway analyses between two subgroups were also performed. All analyses above were performed by R 4.0.2.

### Calculation of Prognostic Risk Scores and Clinicopathological Relativity

Univariate Cox regression was performed to evaluate the correlation between OS or DFS and the transcriptome levels of differentially expressed genes (DEGs) above. The inclusion genes with a cut-off criterion of *p* < 0.05 were then selected for LASSO Cox regression analysis (Friedman et al., 2010; Simon et al., 2011; Tibshirani et al., 2012). Five m^6^A regulators were culled as indispensable biomarkers. Weighed summation of selected biomarkers was calculated and termed risk score, a novel prognostic signature. Then the validity of the risk score was tested by survival analysis, risk plot and receiver operating characteristic (ROC) curve. Univariate and multivariate Cox regression analyses were performed to validate the independent role of the risk signature. The analyses above were performed by R 4.0.2. Finally, the relevance of risk score and clinicopathological features was evaluated by Prism 7.04 (GraphPad Software Inc., La Jolla, CA, United States).

### RNA Extraction and qRT-PCR Assay

Total RNA was isolated from tissues using TRIzol^®^ reagent (Thermo Fisher Scientific, United States). The concentration and purity of the RNA solution were detected using a NanoDrop 2000 spectrophotometer (Thermo Fisher Scientific, United States). Extracted RNA was then reverse transcribed into cDNA using PrimeScript^TM^ RT Master Mix (Takara, Japan) according to the manufacturer’s protocols. The reaction conditions were as follows: 37°C for 15 min; 85°C for 5 s. Subsequently, the cDNA was subjected to qPCR using AceQ^®^ qPCR SYBR Green Master Mix (Vazyme, China) on CFX Connect Real-Time PCR Detection System (Biorad, China) according to the manufacturer’s protocols. The qPCR conditions were as follows: pre-denaturation at 95°C for 5 min; 40 cycles of denaturation at 95°C for 10 s; annealing and extension at 60°C for 30 s. The housekeeping gene, GAPDH, was used to normalize the relative expression of HNRNPA2B1, NXF1, RBMX, YTHDF1, and TRMT112 as an endogenous control by the comparative Ct (threshold cycle) method (2^–ΔΔ*Ct*^). All qRT-PCR reactions were performed in duplicate. The primers used to amplify target genes and GAPDH were chemically synthesized by TSINGKE, China. The primer sequences were listed in Supplementary Table 1.

### Genetic Alterations Indicated Potential Effects of m^6^A RNA Methylation Related Genes in Patients With Prostate Cancer

**FIGURE 1:**
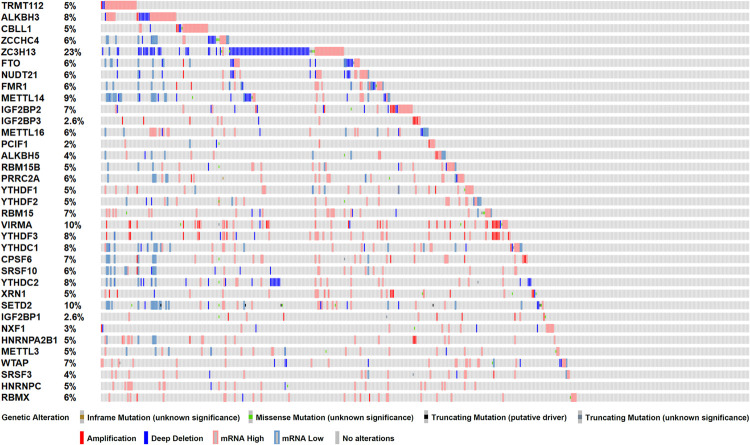
Genetic alterations of m^6^A RNA methylation regulators in TCGA-PRAD cohort (*n* = 499). Mutations include inframe mutation, missense mutation, and truncating mutation. Copy-number alterations (CAN) include amplification and deep deletion.

To investigate the role of m^6^A RNA methylation regulators in patient with PC, we first considered the genome information. The profiles of m^6^A RNA methylation related genetic alteration, including mutation and putative copy-number alteration (CAN), were accessed. Of 499 PRAD patients, 368 (73.7%) harbored at least one type of genetic alterations, including inframe mutation, missense mutation, truncating mutation, amplification, and deep deletion, as well as transcriptomic changes (Figure 1). From the result above, we speculated that significant changes in m^6^A methylation regulators in the genome of PC tissues may lead to that in transcriptome and regulate relevant biological processes.

### Gene Expression of m^6^A RNA Methylation Regulators Jointly Involved in Prostate Cancer Development

**FIGURE 2:**
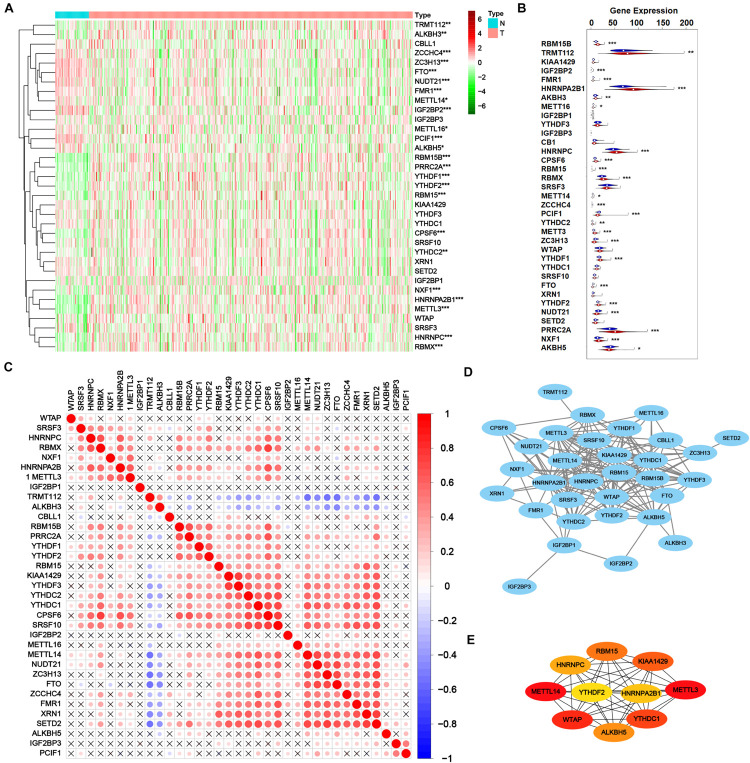
Transcriptome profiles of m^6^A RNA methylation regulators in prostate adenocarcinoma. **(A,B)** The differential expression of m^6^A related genes between 499 tumorous tissues and 52 normal tissues in TCGA-PRAD cohort. **(C)** The correlation of the m^6^A regulatory genes. **(D)** Protein-protein interaction (PPI) network of m^6^A regulatory genes. Elements not connected to other genes are hidden. **(E)** PPI network of top 10 pivotal genes from all m^6^A regulatory genes obtained by CytoHubba plugin of Cytoscape.

Then transcriptome profile of m^6^A RNA methylation regulators was thoroughly investigated. RNA-Seq data from TCGA-PRAD cohort was downloaded, including the data of cancer tissue (*n* = 499) and para-cancer tissue (*n* = 52). The information of m^6^A RNA methylation regulators was extracted and analyzed for DEGs. As shown in Figures 2A,B, 24 of 35 m^6^A regulators expressed differently with the significance of *p* < 0.05, including 14 up-regulated genes (RBM15B, TRMT112, HNRNPA2B1, ALKBH3, HNRNPC, CPSF6, RBM15, RBMX, YTHDC2, METTL3, YTHDF1, YTHDF2, PRRC2A, and NXF1) and 10 down-regulated genes (IGF2BP2, FMR1, METTL16, METTL14, ZCCHC4, PCIF1, ZC3H13, FTO, NUDT21, and ALKBH5), indicating the obvious variation of m^6^A modification in tumorigenesis.

After validating the DEGs related to m^6^A, the interaction among all m^6^A regulators was explored. As shown in Figure 2C, most m^6^A RNA methylation regulators correlated with others. Thereinto, RBMX, HNRNPA2B1, RBM15B, PRRC2A, YTHDF1, YTHDF2, RBM15, KIAA1429, YTHDF3, YTHDC2, YTHDC1, CPSF6, SRSF10, METTL14, NUDT21, ZC3H13, FTO, ZCCHC4, FMR1, XRN1, and SETD2 positively correlated with other regulators while TRMT112 and ALKBH3 negatively correlated. However, some regulators such as WTAP, IGF2BP1, IGF2BP2, IGF2BP3, ALKBH5, and PCIF1 showed poor correlation with other regulators, which may result from their versatility in multiple biological processes. Figure 2D showed a protein-protein interaction (PPI) network of 35 m^6^A methylation regulators, suggesting a close connection between them. A novel algorithm of Maximal Clique Centrality (MCC) of CytoHubba mode in Cytoscape was performed to filter the hub genes of the network based on known or predicted PPIs (Figure 2E). Notably, IGF2BP2 and IGF2BP3 seemed quite isolated from the major network, in accordance with the correlation plot. Yet PRRC2A and ZCCHC4 had no observed connection with other regulators but strongly correlated, which is worth further investigation.

### Consensus Clustering Categorized Patients According to DEGs Related to m^6^A

**FIGURE 3:**
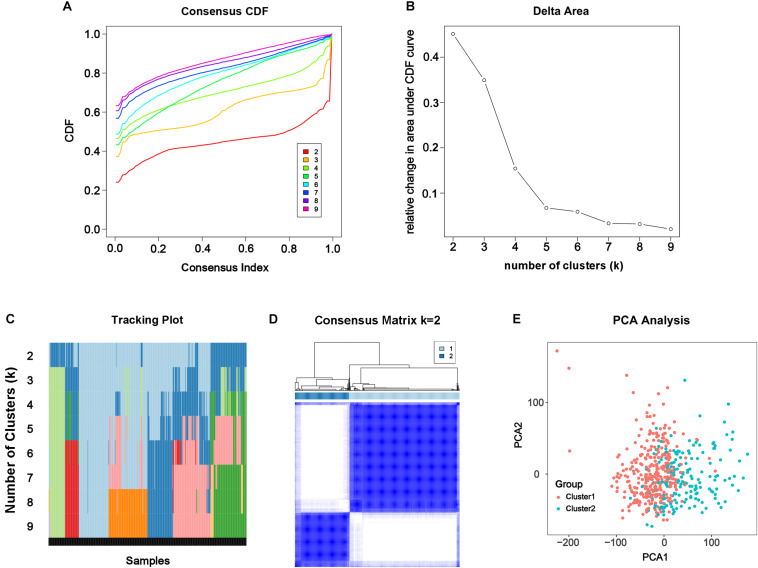
Consensus clustering of the tumorous cohort from TCGA-PRAD based on the differently expressed m^6^A regulatory genes. **(A)** Consensus clustering distribution function (CDF) for *k* = 2 to 9. **(B)** Area under CDF curve increment for *k* = 2 to 9. **(C)** Tracking plot for *k* = 2 to 9. **(D)** Consensus matrix for optimal *k* = 2. **(E)** Principal components analysis (PCA) of the total transcriptomic profile from TCGA-PRAD cohort for optimal *k* = 2.

From the results above, the alteration of m^6^A modification in PC had been confirmed genomically and transcriptomically, but we doubted that it was clinically or biologically meaningful. To investigate the effect of m^6^A RNA methylation regulator in development of PC, consensus clustering was applied to divide the tumorous tissues into subgroups according to the RNA-seq data of 24 differentially expressed m^6^A RNA methylation regulators. Cumulative distribution function (CDF) of the consensus cluster for *k* = 2 to 9 and increment in the AUC were shown in Figures 3A,B. Under two considerations, maximum AUC increment of CDF and expression correlation of m^6^A RNA methylation regulators that is high within groups and low between groups, *k* = 2 was determined, namely the number of clusters (Figure 3D). The tracking plot of subgroups for *k* = 2 to *k* = 9 was shown in Figure 3C. PCA for total transcriptomic data from TCGA-PRAD cohort was executed to access the validity of consensus clustering and offer an intuitionistic sign of two clusters (Figure 3E), which showed distinctly different characteristics of two clusters.

### Clusters Varied in Prognosis and Predicted Function

**FIGURE 4:**
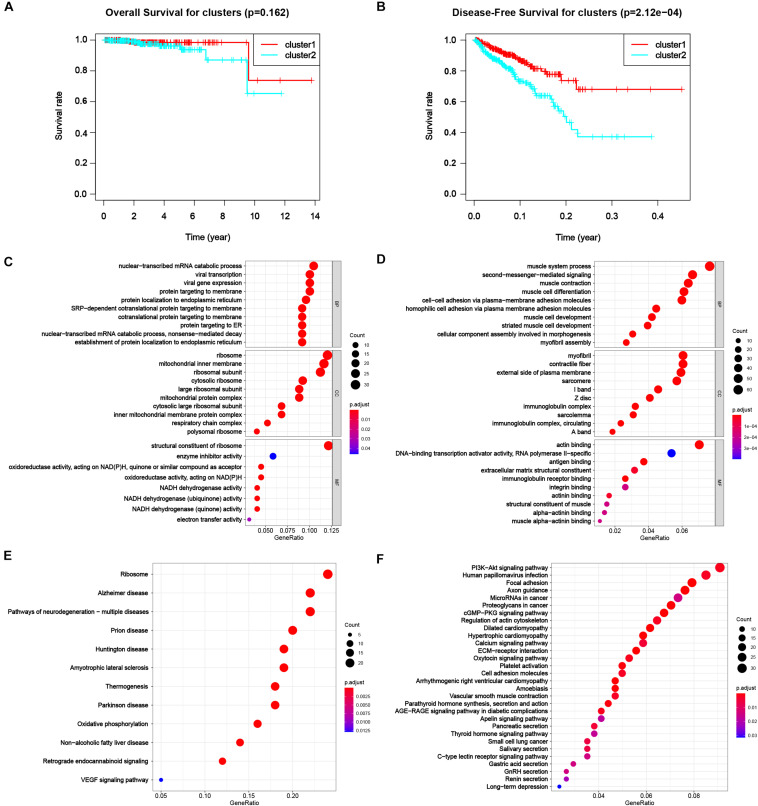
Differential characteristics of total transcriptomic profile in TCGA-PRAD tumorous cohort between clusters. **(A)** Kaplan–Meier overall survival (OS) curves for patients in distinct clusters (*p* = 0.162). **(B)** Kaplan–Meier disease-free survival (OS) curves for patients in distinct clusters (*p* < 0.001). **(C,D)** Gene Ontology (GO) analysis of differentially expressed genes (DEGs) between clusters. Left, up-regulated DEGs; right, down-regulated DEGs. BP, biological process; CC, cellular component; MF, molecular function. **(E,F)** Kyoto Encyclopedia Genes and Genomes (KEGG) analysis of DEGs between clusters. Left, up-regulated DEGs; right, down-regulated DEGs.

To appraise the characteristics of the patients between clusters, the survival analysis was carried out (Figure 4B), suggesting a worse DFS of cluster 2 compared to cluster 1 with the significance of *p* < 0.001. Although the OS of patients in cluster 2 was also worse than that in cluster 1 (Figure 4A), the significance was larger than 0.05, the reason of that might be a dearth of dead cases in the dataset due to high morbidity and low mortality of PC. The results showed a distinguishing classification method by the profiles of differentially expressed m^6^A RNA methylation regulators and offered us a primary impression that alterations in expression profiles of m^6^A related genes affected the prognosis of PC.

Next, we were engrossed in the functional differences between the two subgroups. The result of GO was shown in Figures 4C,D for up-regulated and down-regulated genes, respectively. Up-regulated DEGs highly enriched in processes related to protein biosynthesis, including mRNA catabolic processes, ribosomal activities, protein translocation, and energy transfer. Down-regulated DEGs mostly enriched in muscle-cell related functions, e.g., muscle system process, muscle contraction and muscle cell development and differentiation in biological process (BP), myofibril, contractile fiber, sarcomere I band and Z disc in cellular component (CC), and actin binding in molecular function (MF), that might result from degeneration of smooth muscle in prostate infiltrated by cancer tissue. Also, down-regulated genes enriched in malignancy-associated process, including extracellular matrix organization, cell-cell adhesion via plasma-membrane adhesion molecule and homophilic cell adhesion via plasma membrane adhesion molecules. The results of KEGG were shown in Figures 4E,F for up-regulated and down-regulated genes, respectively. Classical pathways in tumor pathology including PI3K-Akt pathway, focal adhesion and proteoglycans in cancer were all enriched in down-regulated DEGs. Part of the pathways were related to some other diseases, like Alzheimer disease (AD), Huntington disease (HD), and Parkinson disease (PD) for up-regulated DEGs and dilated cardiomyopathy (DCM) and hypertrophic cardiomyopathy (HCM), were also enriched.

### A Novel Risk Signature Was Constructed Based on Five Key m^6^A RNA Methylation Regulators for Prognosis Prediction of Prostate Cancer

As we had figured out the important effect of RNA methylation regulators on the development of PC, we longed to unearth the prognosis value of them in PC. As PPI based on universal data alone could not fully reflect the clinicopathological characteristics of PC patients, we combined the prognosis and transcriptome data of PC patients to screen the critical genes that affect the prognosis of PC patients.

**FIGURE 5:**
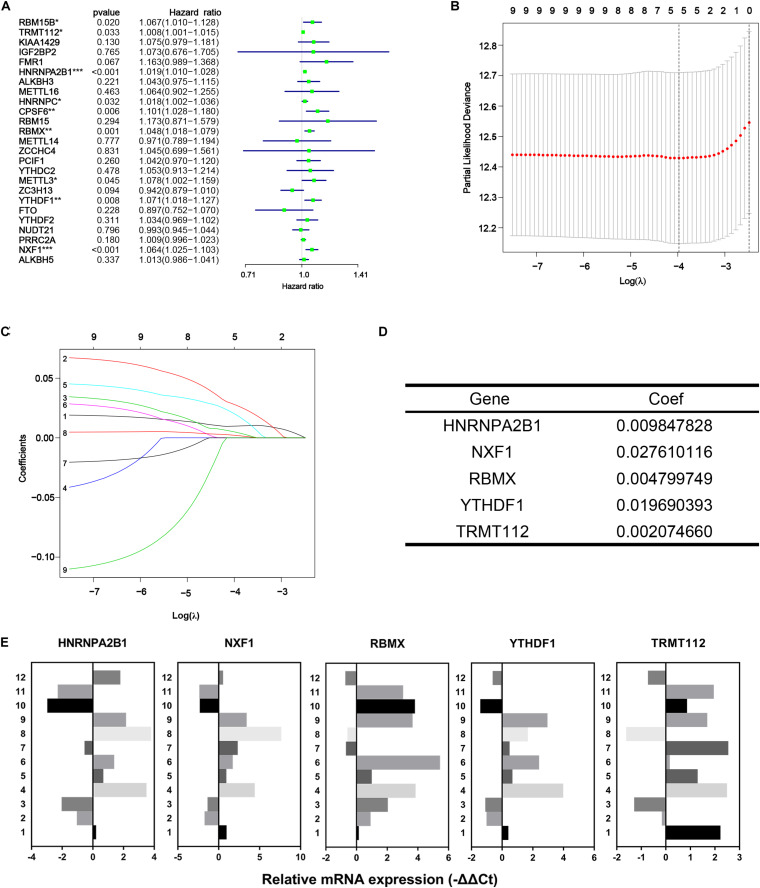
Identification of five critical m^6^A regulatory genes. **(A)** Forest plot of univariate Cox regression analysis for differentially expressed m^6^A regulatory genes. **(B–D)** The last absolute shrinkage and selection operator (LASSO) Cox regression for m^6^A regulatory genes that meet the criteria of *p* < 0.05 in univariate Cox regression analysis. **(E)** Relative mRNA expression of selected m^6^A regulatory genes measured by qRT-PCR assays.

In the first place, univariate Cox regression was performed based on the RNA-Seq data of 25 differentially expressed m^6^A methylation regulators from TCGA-PRAD dataset to appraise the efficacy of prognosis value of individual regulators. As a result, nine of 25 regulators significantly correlated to DFS with *p* < 0.05, including RBM15B, TRMT112, HNRNPA2B1, HNRNPC, CPSF6, RBMX, METTL3, YTHDF1, and NXF1 (Figure 5A). All nine regulators above were risk factors with HR > 1. Then LASSO Cox regression analysis for those nine regulators was performed to make up a comprehensive and effective risk signature for prognosis (see Figure 5B,C). Five critical genes stuck out were HNRNPA2B1, NXF1, RBMX, YTHDF1, and TRMT112 (Figure 5D). Then the weighed summation of gene expression levels of constituent biomarkers, i.e., risk score, for tumorous samples were calculated based on the coefficients determined by LASSO Cox regression.

### Risk Score Based on Five Critical Genes Well Predicted the Prognosis of Prostate Cancer and Associated With Multiple Clinicopathological Features

**FIGURE 6:**
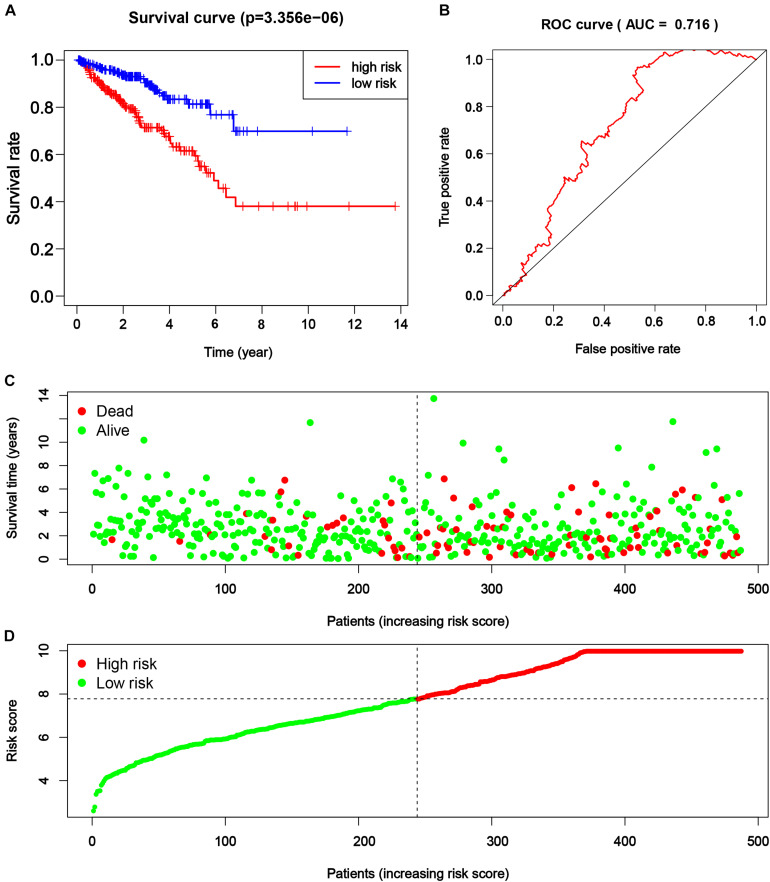
The efficacy of novel risk signature consisted of five m^6^A regulatory genes. **(A)** Kaplan–Meier disease-free survival (DFS) curves for patients with higher and lower risk score (*p* < 0.001). **(B)** Receiver operating characteristic (ROC) curve for patients with higher and lower risk score (*p* < 0.001, AUC = 0.716). **(C,D)** Risk plots for patients with higher and lower risk score.

Consequently, the PRAD patients from TCGA were divided into a higher and lower half according to the risk score for further assessment. The result of survival analysis illustrated that the high-risk group had worse survival status (disease-free vs. recurred/progressed) in comparison with the low-risk group (*p* < 0.001, Figure 6A). A time-dependent ROC curve illustrated the true positive rate versus false positive rate of the prediction with AUC of 0.716 (Figure 6B), suggesting good prediction performance. The risk plot also depicted a reliable prognostic value of risk score (Figures 6C,D).

**FIGURE 7:**
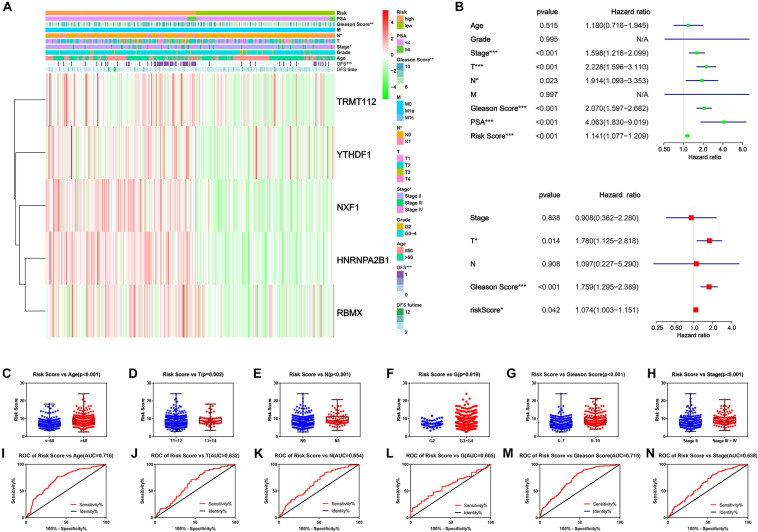
Clinicopathological characteristics of novel risk signature constituted of five m^6^A regulatory genes. **(A)** The heatmap of five constituent genes of risk signature along with clinicopathological characteristics. **(B,C)** Univariate and multivariate Cox regression analyses of risk score along with clinicopathological characteristics. **(C–H)** The distribution of risk score in different clinicopathological characteristics. **(I–N)** Receiver operating characteristic (ROC) curve for patients with different clinicopathological characteristics.

Finally, we explored the connection of risk scores, clinicopathological features and expression levels of critical genes. The heatmap suggested that patients in the high-risk group harbored a significantly higher Gleason score, N stage and AJCC clinical stage (Figure 7A). Afterward, univariate and multivariate analyses were performed for confirmation of an independent prognostic indicator. According to univariate Cox regression, AJCC stage, T stage, N stage, Gleason score, and prostate-specific antigen (PSA) and risk score were significantly associated with DFS (Figure 7B). Multivariate Cox regression confirmed the T stage, Gleason score and risk score as independent prognostic indicators (Figure 7D). Moreover, the risk score inclined with older age and higher clinicopathological stage, including AJCC clinical stage, T stage, N stage, G stage, and Gleason Score (Figures 7D–I). From the above findings we concluded that the novel prognostic signature integrated by five critical m^6^A RNA methylation regulators could independently predict the prognosis of PC.

## Discussion

Prostate cancer is a disease with leading new cases and secondary new deaths among men with cancer. Although PC has a better prognosis compared with many other types of cancers, the negative impact of recurrence, progression, and metastasis on human life span and quality of life should not be underestimated. At present, the major treatment methods for PC are surgery, endocrine therapy, chemotherapy, radiation therapy, cryotherapy, and immunotherapy. In terms of drug therapy, anti-androgen drugs and castration drugs are the mainstreams. However, castration-resistant prostate cancer (CRPC) caused by long-term use of drug is calling for new therapeutic targets.

Previous studies have shown that m^6^A RNA methylation, the most frequent modification in RNA, closely relates to many types of cancers (Ortega et al., 2003; Casalegno-Garduno et al., 2010; Linnebacher et al., 2010; Kaklamani et al., 2011; Pierce et al., 2011; Heiliger et al., 2012; Jin et al., 2012; Machiela et al., 2012; Akilzhanova et al., 2013; Lin et al., 2013; Long et al., 2013; Reddy et al., 2013; Hesser et al., 2018; Tan and Gao, 2018), but its roles in the occurrence and development of PC have not been fully explained. Nowadays, research on the roles of individual genes in tumorigenesis via mediating m^6^A methylation is in full swing. METTL3 promotes development, progression and metastasis of PRAD through regulating MYC methylation, hedgehog pathway, and Wnt pathway (Cai et al., 2019; Ma et al., 2020; Yuan et al., 2020) and enhances bone metastasis through m^6^A-HuR-dependent mechanism (Li et al., 2020). VIRMA sustains invasiveness of PC cell through regulating oncogenic lncRNAs (Barros-Silva et al., 2020). YTHDF2 promotes the proliferation and migration of PC that is suppressed by miR-493-3p (Li et al., 2018). However, we cannot ignore the holistic study of m^6^A RNA modification regulators, which is a virgin territory in PC. On this paper, we studied genes, including some crucial factors, that played major roles in m^6^A methylation modification for guidance of future research directions.

First, we selected 35 important m^6^A-related genes from literatures, finding their significant genetic alterations. Next, we conducted an analysis on TCGA transcription profiles, obtaining 24 DEGs between PC tissue and para-cancer tissue. Afterward, consensus clustering based on their expression levels was conducted to divide the TCGA-PRAD cohort into two clusters. Through PCA and survival analysis, we confirmed the significant discrimination between groups. In addition, GO analysis illustrated the function of DEGs between two clusters: for up-regulated DEGs the enrichment of pathways involved in gene transcription, mRNA translation, and protein translocation, in accordance with the higher activity of m^6^A RNA methylation regulators; for down-regulated DEGs the function involved in muscle contraction-related genes, making prostate smooth muscle cells alter their normal contraction and induce the malignant tendency which provided a new direction for further research. KEGG analysis provided us with even more interesting results: up-regulated DEGs may inextricably linked to many other diseases, including many neurodegenerative diseases, human papillomavirus (HPV) infection, non-alcoholic fatty liver disease (NAFLD); down-regulated DEGs not only affected some classic cancer-related biological processes, such as PI3K-Akt pathway and focal adhesion, but may also participate in the onset of cardiomyopathy. According to the literatures, AD and PC shared genetic etiology via gene expression regulators (Feng et al., 2017), but the answer as to whether a patient harboring AD is a protective factor of PC was controversial (Roe et al., 2005; Ou et al., 2013; Lee et al., 2018; Lin et al., 2018). Many researchers stated that HD or PD patients were less likely to develop PC than healthy people (Jespersen et al., 2016; McNulty et al., 2018). As regards HPV infection, it is recognized as the cause of many cancers, including cervical cancer and anal cancer. Although there stands much evidence to support it as a risk factor of PC, no unified conclusion has been reached yet (Whitaker et al., 2013; Singh et al., 2015; Yang et al., 2015; Moghoofei et al., 2019). Regarding NAFLD, some literatures consider it as a protective factor for PC biochemical recurrence (Choi et al., 2014), but a large sample epidemiological study declared it as a risk factor of PC (Choi et al., 2018). The results above were hints for further exploration of function alteration and linked diseases of PC.

Subsequently, through univariate single Cox regression analysis on 24 DEGs, we obtained nine genes that were significantly associated with survival time. Later, LASSO Cox regression analysis selected five critical genes and constructed an integrated risk score for prognosis prediction of PC patients. All these genes above were confirmed up-regulated in cancer compared to para-cancer by qRT-PCR assays (Figure 5E). Survival analysis, ROC curve, univariate and multivariate Cox regression analyses illustrated that it is a reliable independent prognostic indicator of PC and had a significant relationship with many clinicopathological features. With the promotion of clinical grade and Gleason score, the risk score increased, which not only suggested that the expression of critical genes may promote tumor progression, but also proved the importance of the risk score in predicting the prognosis of PC. At present, most cancer risk scores were based on clinicopathological characteristics, including clinical symptoms, pathology, histology, key bioproteins and metabolites. Also, many studies were exploring the utility of genomics for risk prediction, such as polygenic risk score. However, the role of transcripts, a messenger connecting genes and biomacromolecules, should not be ignored, and risk scores based on them are potentially applicable.

Finally, we aimed to discuss and predict the mechanism by which these critical genes affect the occurrence, development, and prognosis of PC.

HNRNPA2B1, also known as HNR(N)PA2 and HNR(N)PB1, is an RNA binding protein and complex with heterogeneous nuclear RNA (hnRNA). HNRNPA2B1 mostly binds a set of mRNAs in nuclear and elicits alternative splicing effects but it also works in part on primary microRNA (Alarcon et al., 2015). HNRNPA2B1 took part in many biological processes, such as cell survival regulation, cell cycle alteration, telomere maintenance, metastasis regulation, and cellular energetics regulation, and acted as a prognostic biomarker in multiple cancers including lung cancer, pancreas cancer, and hepatocellular cancer (Chen et al., 2017; Dai et al., 2017; Roy et al., 2017; Yu et al., 2018). HNRNPA2B1 also highly expressed in CRPC and associated with tumor progression and prognosis (Cheng et al., 2020). Through quantitative proteomic mass spectrometry profiling, HNRNPA2B1 was likely involved in TGF-β induced-EMT transition of PC (Singh and Sharma, 2020).

NXF1 matters in interaction between two m^6^A readers, YTHDC1 and SRSF3, for mRNA export promotion (Roundtree et al., 2017b). Pitifully, the link between NXF1 and any cancer has not been established.

HNRNPG, also known as RBMX, is an m^6^A reader that preferentially binds non-coding RNAs and also binds m^6^A on mRNAs for their splicing (Zhou et al., 2019). Literatures exist showing that RBMX predicted prognosis in patients with head and neck cancer (Guo et al., 2020) and regulated apoptosis in breast cancer (Martinez-Arribas et al., 2006). The function of RBMX in PC remains unknown.

YTHDF1 is a cytosolic m^6^A reader that preferentially binds m^6^A sites in mRNAs and promotes translation of a subset of m^6^A-containing mRNA (Shi et al., 2018; Zhuang et al., 2019). YTHDF1 served as a prognostic factor in ovarian cancer (Liu et al., 2020), hepatocellular carcinoma (Zhao et al., 2018), lung cancer (Shi Y. et al., 2019), etc. In terms of the function, YTHDF1 regulated tumorigenicity and cancer stem-cell-like activity in colorectal carcinoma (Bai et al., 2019), promoted hypoxia adaption in lung cancer (Shi Y. et al., 2019), promoted ovarian cancer progression, and facilitates oral squamous cell carcinoma tumorigenesis (Zhao et al., 2020). However, the role of YTHDF1 in PC still needs to be clarified.

TRMT112 is a methyltransferase activator which stablishes a key enzyme responsible for 18S rRNA m^6^A modification named METTL5 (van Tran et al., 2019). Together with C21orf127, TRMT112 acutely affected the proliferation of androgen receptor-dependent, as well as that of castration- and enzalutamide-resistant PC cells and xenograft tumors (Metzger et al., 2019).

In summary, our results systematically demonstrate the expression, potential function, and prognostic value of m^6^A RNA methylation regulators in PC. The expression of m^6^A RNA methylation regulator is highly correlated with the malignant clinicopathological features of PC. Our research provides important evidence for further testing the role of m^6^A methylation in PC.

## Data Availability Statement

Publicly available datasets were analyzed in this study. This data can be found at Xena browser (https://xenabrowser.net/), cBioPortal (http://www.cbioportal.org/), and TCGA project (https://portal.gdc.cancer.gov/).

## Author Contributions

JX and YL designed the study. JX, JL, TX, GC, YS, JT, and LL carried out data acquisition and analysis. JX, YL, XZ, and KC wrote the manuscript. JX, WX, ZX, CY, and ZC collected the clinical samples and managed the clinical data. HY and HL contributed to bioinformatics analysis. XZ and KC were involved in project management and contributed to preparing and making figures and tables. KC and XZ supervised the study. All authors read and approved the final manuscript.

## Conflict of Interest

The authors declare that the research was conducted in the absence of any commercial or financial relationships that could be construed as a potential conflict of interest.

## Footnotes

1 https://portal.gdc.cancer.gov/

2 http://www.cbioportal.org/

3 https://xenabrowser.net/

## Abbreviations

M^6^A: N^6^-methyladenosine
TCGA: the Cancer Genome Atlas
LASSO: the last absolute shrinkage and selection operator
ROC: receiver operating characteristic
PC: prostate cancer
PRAD: prostate adenocarcinoma
PCA: principal components analysis
GO: Gene Ontology
KEGG: Kyoto Encyclopedia Genes and Genomes
AJCC: American Joint Committee on Cancer
OS: overall survival
DFS: disease-free survival
DEG: differentially expressed gene
CAN: copy-number alteration
PPI: protein-protein interaction
MCC: Maximal Clique Centrality
CDF: cumulative distribution function
BP: biological process
CC: cellular component
MF: molecular function
AD: Alzheimer disease
HD: Huntin gton disease
PD: Parkinson disease
DCM: hypertrophic cardiomyopathy
HCM: hypertrophic cardiomyopathy
PSA: prostate-specific antigen
CRPC: castration-resistant prostate cancer
HPV: human papillomavirus
NAFLD: non-alcoholic fatty liver disease.
