# Supplementary material for: The Identification of Critical m6A RNA Methylation Regulators as Malignant Prognosis Factors in Prostate Adenocarcinoma
Source: Front Genet. 2020 Dec 4;11:602485. doi: 10.3389/fgene.2020.602485 (PMC7746824; doi:10.3389/fgene.2020.602485)
Supplement: Supplementary file 1 [file Table_1.DOCX]

**Supplementary files for “The Identification of Critical m6A RNA Methylation Regulators as Malignant Prognosis Factors in Prostate Adenocarcinoma”**

Jiaju Xu^1†^, Yuenan Liu^1†^, Jingchong Liu^1^, Tianbo Xu^1^, Gong Cheng^1^, Yi Shou^1^, Junwei Tong^1^, Lilong Liu^1^, Lijie Zhou^1^, Wen Xiao^1^, Zhiyong Xiong^1^, Changfei Yuan^1^, Zhixian Chen^1^, Di Liu^1^, Hongmei Yang^2^, Huageng Liang^1^, Ke Chen^1*^ and Xiaoping Zhang^1*^

^1^Department of Urology, Union Hospital, Tongji Medical College, Huazhong University of Science and Technology, Wuhan 430022, Hubei Province, China

^2^Department of Pathogenic Biology, School of Basic Medicine, Huazhong University of Science and Technology, Wuhan 430030, Hubei Province, China

†**These authors contributed equally to this work.**

***Correspondence:**

Ke Chen

shenke@hust.edu.cn;

Xiaoping Zhang

xzhang@hust.edu.cn.

**Table S1 Primers used in qRT-PCR**

| Gene | Forward Primer (5'-3') | Reverse Primer (5'-3') |
| --- | --- | --- |
| HNRNPA2B1 | ATTGATGGGAGAGTAGTTGAGCC | AATTCCGCCAACAAACAGCTT |
| NXF1 | GACGAGGGGAAGTCGTACAG | CCAGACCTACGGTTTCCTTCA |
| RBMX | CTTCAGGACCAGTTCGCAGTA | TCACGACCACTTGAGTAGAGAT |
| YTHDF1 | ACCTGTCCAGCTATTACCCG | TGGTGAGGTATGGAATCGGAG |
| TRMT112 | GGTCCGTATCTGCCCTGTG | GGATCAGACGCAAGTTATCGG |

**
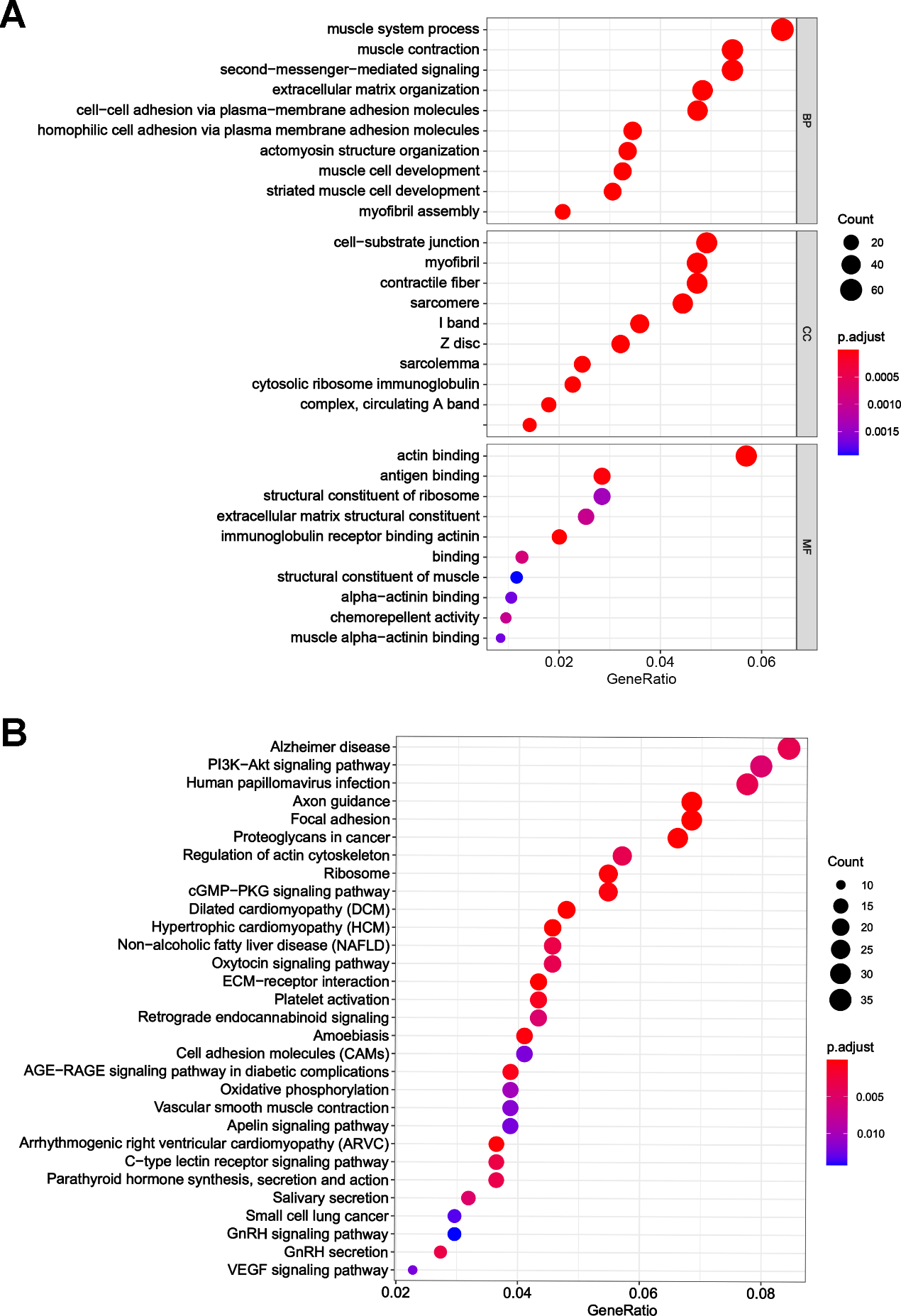
**

**Figure S1 Gene Ontology (GO) and Kyoto Encyclopedia Genes and Genomes (KEGG) analysis for up-regulated DEGs between clusters.**
